# Patterns and risk of HIV-1 transmission network among men who have sex with men in Guangxi, China

**DOI:** 10.1038/s41598-020-79951-2

**Published:** 2021-01-12

**Authors:** Xianwu Pang, Hui Wei, Jinghua Huang, Qin He, Kailing Tang, Ningye Fang, Xinjuan Zhou, Qiuying Zhu, Xiuling Wu, Guanghua Lan, Zhiyong Shen, Mei Lin, Shujia Liang

**Affiliations:** 1 Guangxi Zhuang Autonomous Region Center for Disease Prevention and Control, No. 18 Jinzhou Road, Nanning, 530028 Guangxi China; 2 Health Commission of Guangxi Zhuang Autonomous Region, Nanning, Guangxi China

**Keywords:** Genetics, Microbiology, Molecular biology

## Abstract

The prevalence of HIV-1 in Guangxi is very high, and the rate of HIV-1 infection among men who have sex with men (MSM) has been increasing. Therefore, it is necessary to explore the patterns and risk factors of HIV transmission in Guangxi. For this purpose, individuals diagnosed with HIV-1 during 2013–2018 in Guangxi were recruited. Phylogenetic relationship, transmission clusters, and genotypic drug resistance analyses were performed based on HIV-1 *pol* sequences. Related factors were analysed to assess for their association with HIV-1 transmission. CRF07_BC (50.4%) and CRF01_AE (33.4%) were found to be the predominant subtypes. The analysed 1633 sequences (50.15%, Guangxi; 49.85%, other provinces) were segregated into 80 clusters (size per cluster, 2–704). We found that 75.3% of the individuals were in three clusters (size ˃ 100), and 73.8% were high-risk spreaders (links ≥ 4). Infection time, marital status, and subtype were significantly associated with HIV-1 transmission. Additionally, 80.2% of recent infections were linked to long-term infections, and 46.2% were linked to other provinces. A low level of transmitted drug resistance was detected (4.8%). Our findings indicated superclusters and high-risk HIV-1 spreaders among the MSM in Guangxi. Effective strategies blocking the route of transmission should be developed.

## Introduction

Human immunodeficiency virus (HIV)/acquired immunodeficiency syndrome (AIDS) continues to be among the most important threats to public health in China. Because of the large influence of traditional culture on society, homosexuality is stigmatized and people who are homosexual experience discrimination in China^1,2^. Men who have sex with men (MSM) are inclined to hide their sexual identity and engage in sexual behaviors in other regions rather than in their hometowns, where they may be easily recognized by acquaintances^1–3^. Thus, methods in addition to surveillance and other traditional epidemiological tools are needed to understand the characteristics of HIV-1 transmission among MSM. Previous studies showed that molecular analyses can be used to reliably estimate probable HIV-1 transmission networks^4–6^.

Guangxi, southwest China and adjacent to Vietnam, Yunnan, and Guangdong, is an area heavily affected by HIV-1 in China^7^. Since the first HIV-infected individual was reported in 1996 in Guangxi^8^, the number of infected individuals has greatly increased. By the end of 2018, the number of people living with HIV/AIDS in Guangxi exceeded 80,000, accounting for one-third of cases in China. There are no reports of the HIV-1 transmission network among MSM in Guangxi. In this study, we explored the molecular epidemiological characteristics and genetic transmission network of HIV-1 among MSM in Guangxi by molecular analyses, which will be useful for implementing HIV intervention strategies.

## Methods

### Ethics statement

The study was approved by the Ethics Review Committee of Guangxi Autonomous Region Center for Disease Prevention and Control, and all the methods in this study were performed in accordance with the approved guidelines. All the participants provided their written informed consent to participate in the study.

### Study subjects

All the MSM who were diagnosed with HIV-1 at voluntary counselling and testing clinics in Guangxi, China from January 2013 to December 2018, and who had not been treated with antiviral therapy were enrolled in the study (in total, 955 individuals). A total of 732 sequences were successfully sequenced. In addition, 887 individuals (835 heterosexuals, 45 intravenous drug users, and 7 mother-to-child cases) diagnosed with HIV-1 in Guangxi were included in the study. We performed a BLAST search by using the sequences and selected the top 5 sequences showing the highest homology from publicly available HIV databases at Los Alamos National Laboratory. A total of 1119 reference sequences were selected for analysis after excluding repeated sequences. Overall, 2738 sequences were included for subsequent analysis^9^.

### HIV-1 LAg-Avidity enzyme immunoassay (EIA)

These individuals who had been treated with antiretroviral therapy (ART) and CD4^+^ T cell < 200 were excluded for HIV-1 LAg-Avidity EIA. An HIV-1 LAg-Avidity EIA was performed according to the manufacturer’s instructions (Sedia, Portland, OR, USA). Test specimens were initially run as single samples. If the normalized OD (ODn) was > 2.0, the specimen was classified as a long-term infection. Specimens with an ODn < 2.0 were tested again in triplicate to confirm the obtained values. In confirmatory testing, specimens with an ODn < 1.5 were classified as recent infections.

### Phylogenetic analysis

All sequences were edited by using Sequencher5.1 software and aligned by using BioEdit 7.1 software. The reference sequences were obtained from the Los Alamos National Laboratory database and covered the major HIV-1 subtypes and circulating recombinant forms (CRFs). Phylogenetic tree analyses were performed using the neighbor-joining method based on the Kimura2-parameter model with 1000 bootstrap replicates by using MEGA7.0 software^10^.

### Drug resistance analysis

The sequences of the *pol* gene were submitted to the Stanford HIV Drug Resistance Database to identify drug-related mutations. The degree of drug resistance to each antiretroviral drug was divided into five levels: susceptible, potential low-level resistance, low-level resistance, intermediate resistance, and high-level resistance, according to the HIVDB Genotypic Resistance Test Interpretation System^11^.

### Network construction

These aligned sequences were entered into the HyPhy software to calculate their genetic distances, and Tamura-Nei93 pairwise genetic distances were calculated for all pairs of sequences. Two sequences showing a genetic distance of ≤ 1.5% were identified as potential transmission partners. The network data were processed using Cytoscape v3.5.1 software to construct the transmission network. We determined the characteristics of the network, including nodes (individuals in the network), edges (the link between two nodes, representing the potential transmission relationship between the two individuals), degrees (the number of edges linking one node to other nodes), network sizes (the number of individuals in a cluster), and clusters (groups of linked sequences)^12^. The network data was visualized using a custom R script with the network package in R version 4.0.2 software^13^.

### Statistical analysis

To explore factors associated with a potential transmission, the edge between the two nodes was calculated. Comparisons among individuals were based on the following factors: year diagnosed, domicile region, age, marital status, educational level, ethnicity, HIV subtype, and when infection occurred. Significant differences in categorical variables were analyzed using chi-square or Fisher’s exact tests. *P *values < 0.05 indicated statistical significance. All statistical analyses were performed using the SPSS 21.0 software (IBM, Chicago, IL, USA).

## Results

### Demographic characteristics of the study participants

Among the collected samples, 732 were successfully sequenced. These samples had an average age of 28.11 ± 8.70 years, with 79.92% (585/732) < 35 years. Most participants were of Han (58.2) and Zhuang (35.38%) ethnicity, and 96.99% (710/732) were Guangxi residents. Approximately half (51.78%, 379/732) had a college or higher level of education. Most subjects were single (83.20%, 609/732).

### Distribution of HIV-1 genotypes

**Figure 1:**
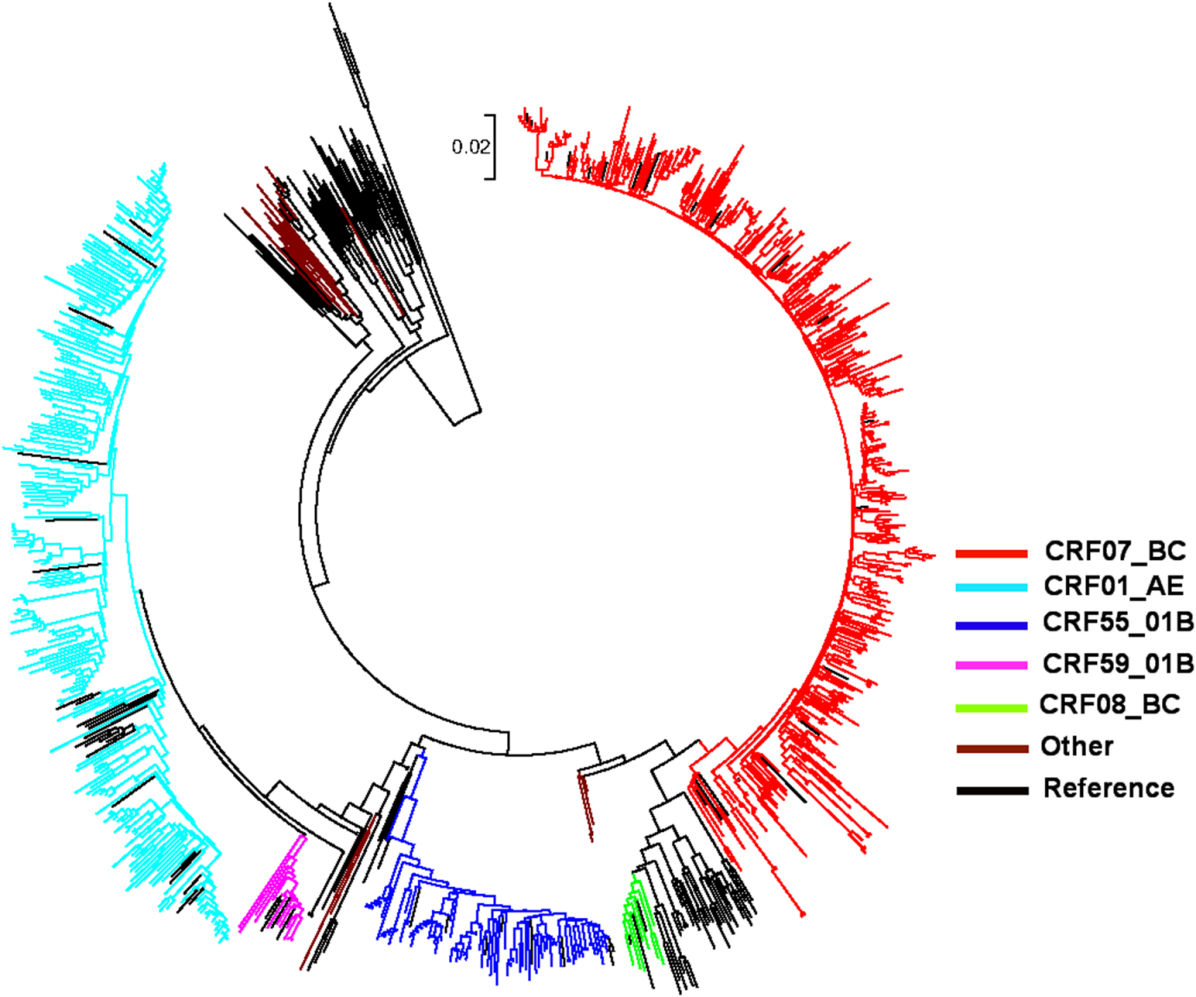
Phylogenetic analysis of the lineages. Phylogenetic tree based on the *pol* region was constructed using the Neighbour-Joining method and the MEGA7.0 software. The various subtypes are colour-coded.

Among the study subjects, 732 (76.6%) samples were successfully sequenced and genotyped. Neighbor–Joining phylogenetic tree analysis performed using MEGA 7.0 revealed that CRF07_BC was the dominant subtype (50.4%, 369/732), followed by CRF01_AE (33.4%, 245/732), CRF55_01B (11.3%, 83/732), CRF59_01B (1.5%, 11/732), subtype B (1.2%, 9/732), CRF08_BC (0.9%, 7/732), CRF67_01B (0.2%, 2/732), CRF68_01B (0.1%, 1/732), subtype A (0.1%, 1/732), and unique recombinant form (URFs; 01_AE/CRF07_BC, CRF01_AE/C) (Fig. 1).

### Identification and characteristics of transmission networks

**Figure 2:**
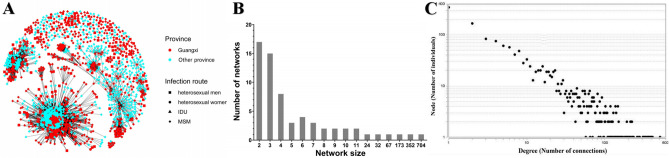
HIV-1 transmission clusters. The network was constructed using a custom R script with the network package in R version 4.0.2 software. (**A**) Infection route and region-associated transmission clusters. Different colours represent different provinces. Different shapes represent different infection routes: solid square: heterosexual men; solid circle: heterosexual women; solid triangle: IDU; solid diamond: MSM. (**B**) Size of HIV-1 transmission clusters among the MSM. (**C**) Distribution of HIV-1 transmission degree. Node represents individuals in the network; degree represents the number of edges that link one node to other nodes; size represents the number of individuals in a cluster.

An HIV-1 transmission network was constructed and is shown in Fig. 2A. Of the 2738 subjects evaluated, 1633 (59.6%) segregated into 80 networks, of which 819 (50.15%) were from Guangxi, and 814 (49.85%) were from other provinces. Among the infection routes, 89.2% of cases occurred among MSM, 8.9% among heterosexual men, 1.2% among heterosexual women, and 0.5% among injection drug users (IDUs). In the network, 611 Guangxi MSM were included, and cluster sizes for this group ranged between 2 and 704 (Fig. 2B). Links (degrees) between each (node) for the 611 Guangxi MSM subjects (nodes) ranged from 1 to 508 and 543 (88.9%) had more than 2 degrees, indicating that each subject was linked to two or more subjects. Additionally, 451 (73.8%) subjects had more than 4 degrees and were considered high-risk spreaders and more likely to spread HIV-1 to other uninfected people. One subject was linked to 508 subjects, classifying this individual as a super spreader (Fig. 2C).

To explore factors associated with a potential transmission, we investigated the influence of the year diagnosed, residence region, age, marital status, educational level, ethnicity, subtype, and when infection occurred on the number of links in the network. A total of 723 Guangxi MSM, newly diagnosed between 2013 and 2018, were included in the analysis. Chi-square test (Table 1) showed that there was a significant difference in the number of links between marital status (*P* = 0.009), subtype (*P* < 0.001), and when infection occurred (*P* < 0.001). Single subjects had more edges than divorced or widowed subjects. Among the different subtypes, CRF55_01B occurred most frequently, followed by CRF07_BC and CRF01_AE. Recent infection was more common than long-term infection.

**Table 1:** Factors associated with potential transmission links (N = 732).

Characteristics	Total	0 Link, n (%)	1 Link, n (%)	≥ 2 Link2, n (%)	*χ*^2^	*P*
**Years of diagnosis**					2.1	0.350
2013–2015	155	30 (19.4%)	10 (6.5%)	115 (74.2%)		
2016–2018	577	96 (16.6%)	57 (9.9%)	424 (73.5%)		
**Region**					2.269	0.322
Locals	710	120 (16.9%)	64 (9.0%)	526 (74.1%)		
Migrants	22	6 (27.3%)	3 (13.6%)	13 (59.1%)		
**Age**					1.205	0.548
< 35 years	583	100 (17.2%)	50 (8.6%)	433 (74.3%)		
≥ 35 years	149	26 (17.2%)	17 (11.4%)	106 (71.1%)		
**Marital status**					13.433	0.009
Singlehood	609	103 (16.9%)	49 (8.0%)	457 (75.0%)		
Married	91	15 (16.5%)	17 (18.7%)	59 (64.8%)		
Divorced or widowed	32	8 (25.0%)	1 (3.1%)	23 (71.9%)		
**Educational level**					5.508	0.480
Junior high school and below	128	30 (23.4%)	9 (7.0%)	89 (69.5%)		
High school or technical school	214	36 (16.8%)	23 (10.7%)	155 (72.4%)		
College and above	379	58 (15.3%)	34 (9.0%)	287 (75.7%)		
Unknown	11	2 (18.2%)	1 (9.1%)	8 (72.7%)		
**Ethnicity**					8.647	0.071
Han	426	64 (15.0%)	33 (7.7%)	329 (77.2%)		
Zhuang	259	55 (21.2%)	27 (10.4%)	177 (68.3%)		
Others	47	7 (14.9%)	7 (14.9%)	33 (70.2%)		
**Subtype**					42.08	< 0.001
CRF01_AE	245	50 (20.4%)	29 (11.8%)	166 (67.8%)		
CRF07_BC	369	52 (13.9%)	29 (7.9%)	288 (78.3%)		
CRF55_01B	83	9 (9.8%)	3 (3.7%)	71 (86.6%)		
Others	35	15 (45.9%)	6 (16.2%)	14 (37.8%)		
**Infection time**^a^					122.186	< 0.001
Recent infection	106	21 (19.8%)	15 (14.2%)	70 (66.0%)		
Long-term infection	363	237 (55.0%)	77 (19.6%)	49 (25.4%)		

^a^Only 469 newly diagnosed MSM in Guangxi from 2016 to 2018 were tested with the LAg-Avidity EIA to classify recent HIV-1 infections.

### The geographic dimension of HIV-1 transmission

**Figure 3:**
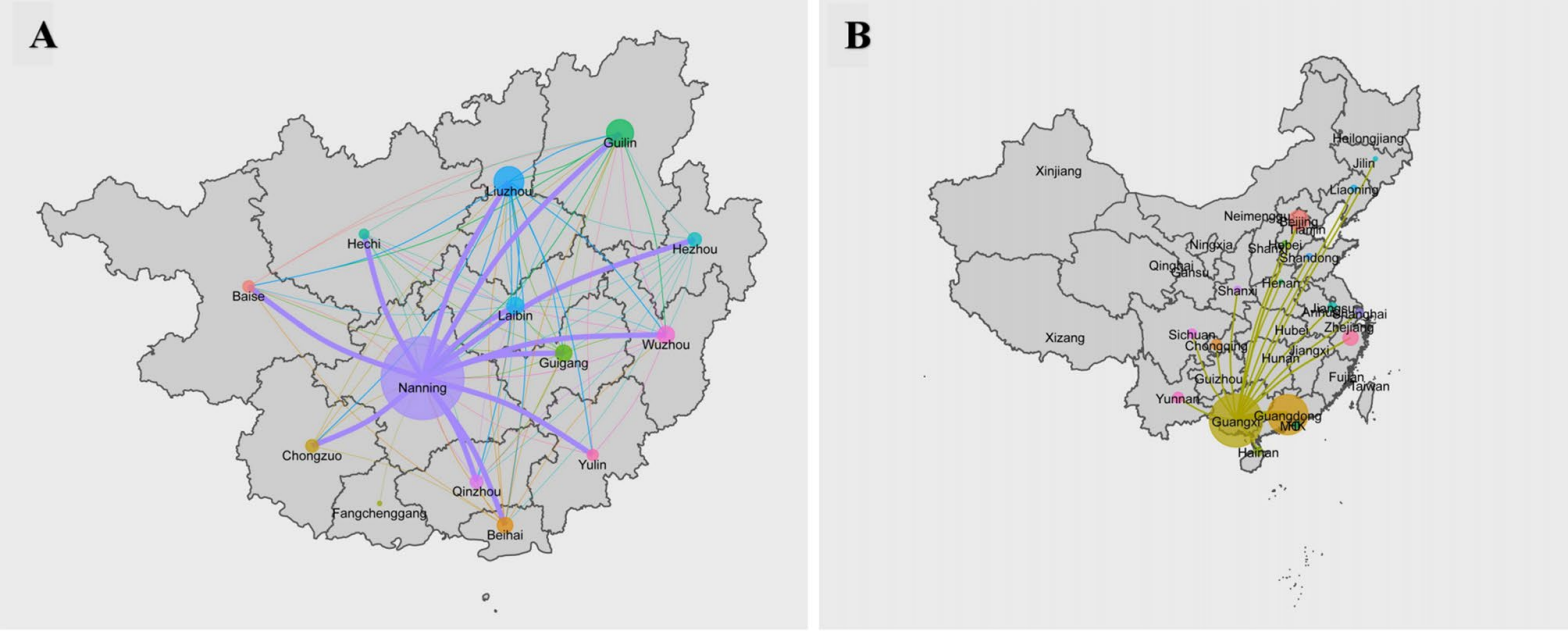
The geographic dimension of HIV-1 transmission. (**A**) HIV-1 transmission in Guangxi. (**B**) HIV-1 transmission between Guangxi and other provinces. Circles with different colours represent the number of individuals; the lengths of the lines represent the degree.

To explore the geographic dimension of HIV-1 transmission among MSM, we constructed transmission routes using the R language. Among all the 14 cities in Guangxi, Nanning (74.4%) showed the largest number of MSM, followed by Liuzhou (7.3%), Guiling (5.8%). Interestingly, Nanning exhibited linkage with the other 13 cities (Fig. 3A). Nationwide, Guangxi was linked with 17 provinces, including Guangdong (57.7%), Beijing (17.9%), Zhejiang (6.4%), Yunnan (5.3%), and others (12.7%) (Fig. 3B).

### Risk factors of recently infected individuals

**Figure 4:**
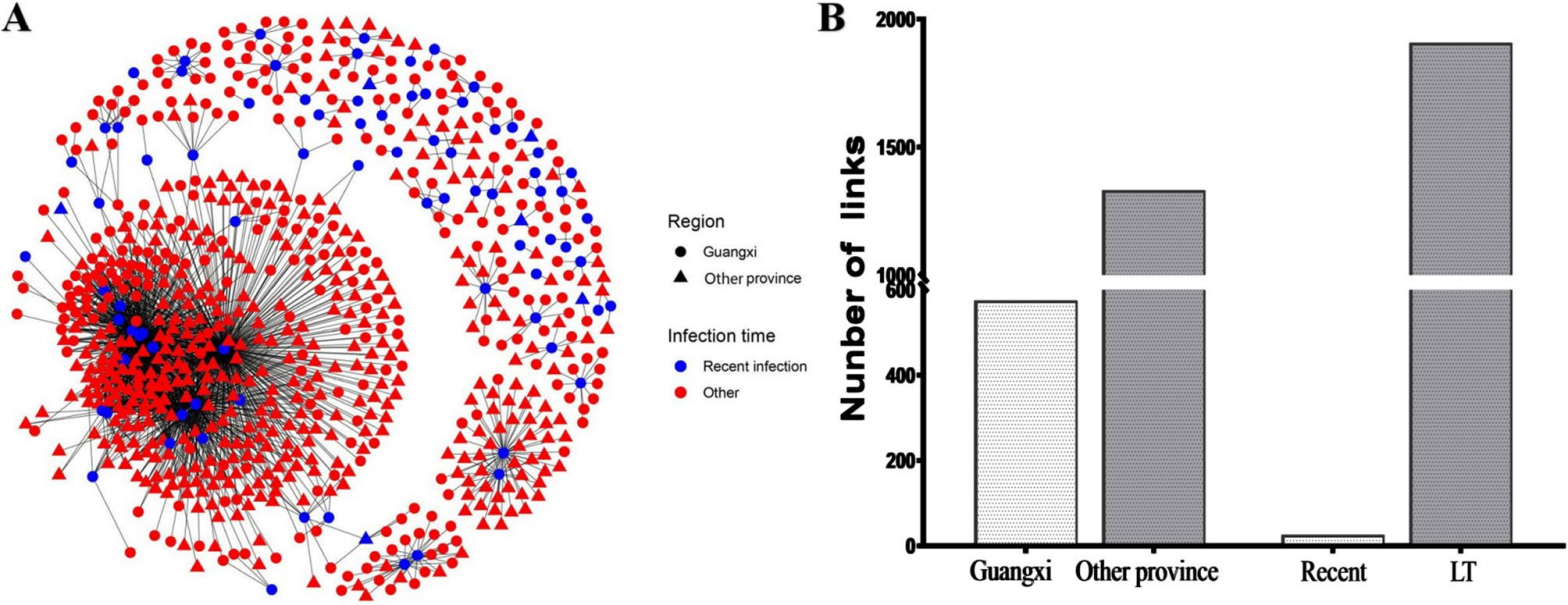
Risk factors of recently infected individuals. The network was constructed using a custom R script with the network package in R version 4.0.2 software. (**A**) Recently infected individuals in transmission networks. Different shapes represent different provinces: solid circle: Guangxi; solid triangle: other provinces. Different colours represent different infection time. Blue, recent infection; red, long-term infection. (**B**) The total number of the links of recent infections in Guangxi with other infections from different areas or for different infection times. *Recent* recent infection, *LT* long-term infection.

To explore the potential transmission patterns between the recently infected subjects and those with long-term infection in the networks according to geographic locations, 106 (of 469, 22.6%) recent infections were identified by performing an LAg-Avidity EIA, of which 26 (24.5%) were linked to other recent infections, and 85 (80.2%) were linked to the long-term infections. 70 (66.0%) were interlinked in Guangxi, 49 (46.2%) were linked to another province (Fig. 4A). To better understand the potential risk of HIV-1 transmission between recent infection and long-term infection, we compared the links between the two. The results showed that recently infected individuals had significantly more links than those with long-term infection (mean of the links, 23.06 vs 1.89), and most of the recent infections had more links with other provinces and long-term infections (Fig. 4B). There was a significant difference in the number of links between recent infection and long-term infection (Table 1).

### Drug resistance-associated transmission networks

**Figure 5:**
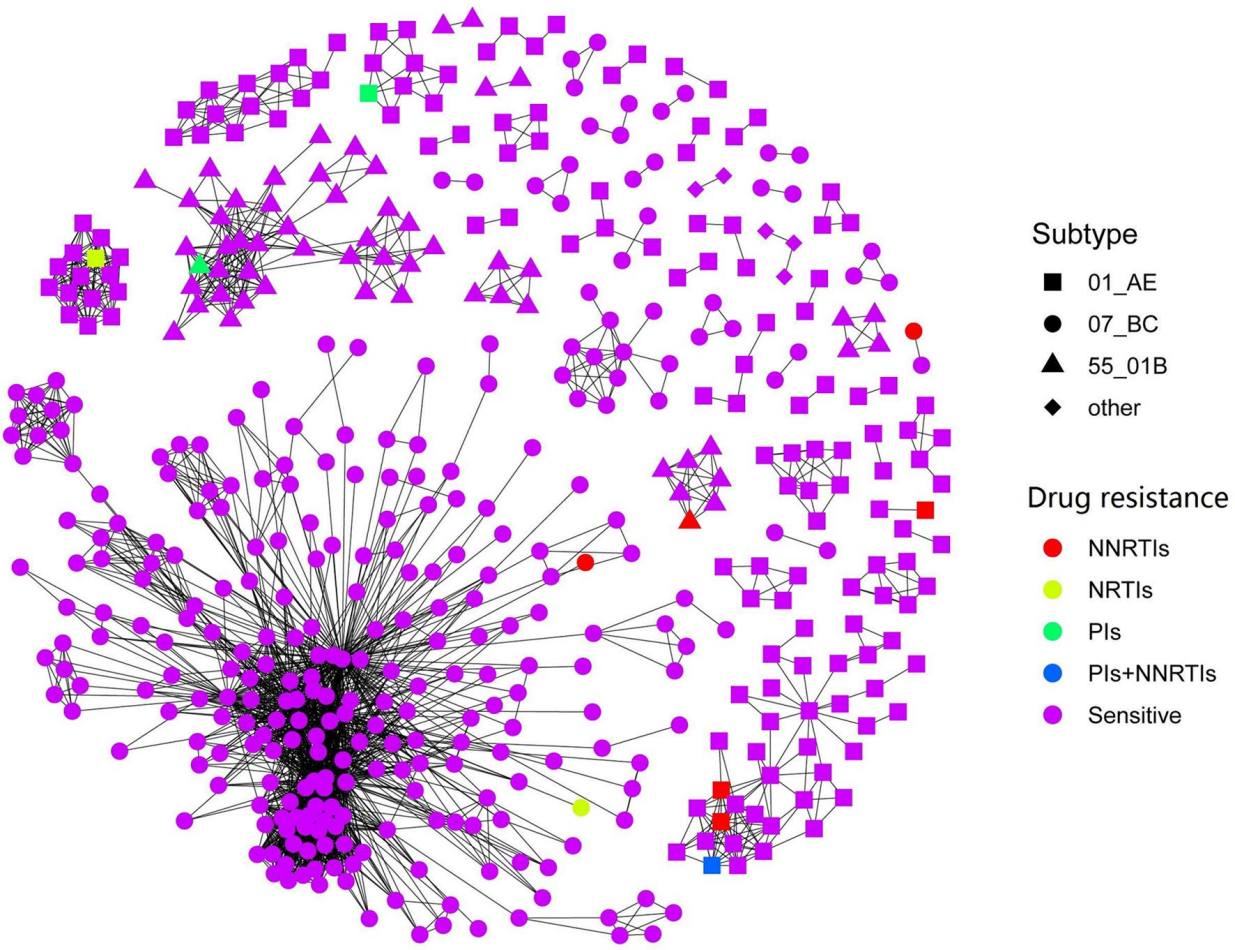
Drug-resistance-associated genetic transmission networks. The network was constructed using a custom R script with the network package in R version 4.0.2 software. Different shapes represent different subtypes. Different colours represent differences in drug resistance. *PIs* protease inhibitors, *NRTIs* nucleoside reverse transcriptase inhibitors, *NNRTIs* non-nucleoside reverse transcriptase inhibitors.

Among the 732 MSM, 35 (4.8%) harboured drug resistance mutations to at least one type of antiretroviral drug. There was no significant difference in the rate of drug resistance between the sampling years (from before 2016 to 2018, 5.2%, 5.1%, 3.8%, 4.9%, respectively, *P* = 0.441). Of all drug resistance-related mutations, 10 (28.6%) were for PIs (protease inhibitor), 4 (11.4%) for NRTIs (nucleoside reverse transcriptase inhibitor), 17 (48.6%) for NNRTIs (non-nucleoside reverse transcriptase inhibitor), 2 (5.7%) for PIs and NNRTIs, 1 (2.9%) for NRTIs and NNRTIs, and 1 (2.9%) for PIs, NRTIs, and NNRTIs. The highest rate of drug resistance was observed for subtype B (55.6%), followed by CRF55_01B (6.0%), CRF01_AE (4.9%), and CRF07_BC (3.5%). Eleven (29.7%) individuals harboured drug resistance mutations that were included in the transmission networks, and three were interlinked, indicating potential transmitted drug resistance (Fig. 5). These individuals with drug resistance in the transmission networks all corresponded to long-term infection.

## Discussion

Our findings show that CRF07_BC and CRF01_AE were the major HIV-1 subtypes among the MSM in Guangxi, which agrees with the results of previous studies^14–18^. CRF07_BC was first found among IDUs in Guangxi in 2002^19^, and gradually formed a large-scale epidemic in the area in recent years. In 2013, CRF01_AE (62.0%), CRF07_BC (25.0%), and CRF08_BC (6.5%) remained as the major strains^20^, with CRF07_BC accounting for 50.4% of cases in MSM in this study. These results suggest that CRF07_BC spread much easier among MSM and became a predominant strain in the region. CRF55_01B was first identified in Chinese MSM in 2012^21^, and a recent paper has reported that CRF55_01B was identified in the MSM in 7 provinces with prevalence rates of 1.5–12.5%^22^. In that study, CRF55_01B accounted for 11.4% in MSM, with CRF55_01B widely disseminated among the MSM in the region. In addition to the major CRFs, we identified 2 CRFs (CRF67_01B, CRF68_01B) that were imported from outside of the province and 2 URFs (01_AE/CRF07_BC, CRF01_AE/C). A previous study reported that higher rates of dual-variant and multiple-variant HIV infection were found in MSM compared to in heterosexual individuals in the same populations^23,24^, increasing the probability of HIV-1 recombination. There is a growing concern that URFs may be prone to be formed in MSM because of the high-risk behaviour features of this population, including multiple sex partners, low rates of condom use, and anal intercourse.

It is challenging to trace the most probable infection route. We used novel molecular methods to explore the probable HIV-1 transmission network and risk associated with potential transmission. We showed that most HIV-1 infections were associated with cases outside of the province, and the main infection route was MSM. Moreover, the HIV-1 transmission network is concentrated; 3 networks contained more than 100 individuals, with the largest network containing 704 individuals. The finding reflects the existence of an HIV-1 super transmission network in the MSM in Guangxi and the characteristics of HIV-1 agglutinative transmission, which differs from those in other provinces of China^9,17^. Further analysis showed that 88.9% of individuals had more than 2°, meaning each subject was linked to at least two subjects; 451 (73.8%) subjects had more than 4° and were considered high-risk spreaders and more likely to spread HIV-1. One individual had 508° and was considered a super spreader. The finding reveals the existence of HIV-1 multiple partners and a super transmission network among MSM in Guangxi. Previous studies showed that individuals with more links in the network have a higher probability of spreading the virus to others because of their high viral load and high rate of partner change; therefore, these individuals may function as super-spreaders^25,26^. Chi-square test shows that marital status, subtype, and infection time were factors associated with potential transmission in networks, with a single status, CRF55_01B, and recent infection more likely to be associated with the virus spreading. This finding reveals why CRF55_01B has been widely disseminated among the MSM in the region in recent years. Thus, additional molecular epidemiology surveillance of CRF55_01B is required.

It is very important to analyze the geographic dimension of HIV-1 transmission among MSM within the province and throughout the country. Guangxi is located in southwest China and is adjacent to Vietnam, Yunnan, and Guangdong. This area of China is heavily affected by HIV-1^7^. Nanning, the capital and largest city of Guangxi, is the provincial center in terms of the economy, culture, science, education, and tourism. Our findings revealed that Nanning is a major region of HIV-1 transmission, and the virus has spread to 13 other cities in Guangxi. Further analysis revealed that Nanning accounted for the dominant proportion of infections, not only in terms of local transmission (74.4%) but also in terms of cross-regional HIV-1 transmission (73.5%) based on the provincial transmission network, highlighting its remarkable role in the intra-provincial spread of HIV-1. The major province of cross-regional HIV-1 transmission with Guangxi is Guangdong, accounting for 57.5% of cases. Guangdong, a prosperous Chinese province adjacent to Guangxi, and it takes approximately 3 h to travel between Guangxi and Guangdong by high-speed railway. In the future, Guangdong is expected to become a major source of HIV-1 transmission. We also found that although most recent infections were associated with long-term infections, recent infections were closely related to potential transmission among networks, which is consistent with the results of previous studies^18,27^. This suggests that early HIV-1 infection plays an important role in transmission, emphasizing the need for early diagnosis and timely antiretroviral treatment.

Our findings revealed an estimated prevalence rate of drug-resistant HIV-1 strains of 4.8% among antiretroviral treatment-naïve MSM in Guangxi, representing a low level of TDR, which is consistent with the results of a previous study in the region^28^ and those of the studies in other regions of China^9,29,30^. However, the rate of drug resistance remains close to a moderate level according to the World Health Organization categorization method. Although it is not necessary to make large adjustments to current antiretroviral therapy regimens, further studies are essential for monitoring TDR.

This study has several limitations. First, most of the sequences from the other provinces were downloaded from the internet, which may not fully reflect the actual situation in the corresponding provinces. However, the results provide direction, which may strengthen regional cooperation. This is especially important in Guangdong, which is the province with the most cross-regional HIV-1 transmission with Guangxi. Second, although our findings show that MSM is the largest factor in HIV transmission, the sample sizes for other routes of infection, including among heterosexuals, were low. Third, a potential sampling bias; we could analyze only the samples that had been diagnosed, but those that had been infected but not diagnosed could not be included in the analysis. Another limitation is that LAg-Avidity cannot be completely accurate to distinguish recent infection and long-term infection, including viral load will be good to define recent infection. In the future, more samples will be included in the genetic transmission network.

We showed that network analysis based on a molecular approach can be used to trace the most probable HIV-1 transmission routes and infer additional features of an HIV-1 epidemic. Additionally, we determined the characteristics and risk of HIV-1 transmission among the MSM in Guangxi. These results can be used to design and implement evidence-based interventions. Further studies are required to determine how to combine the results of network analysis with public health approaches to enable effective HIV-1 intervention.

## Data Availability

The datasets used and analysed during the current study are available from the corresponding author on reasonable request.
